# Exploiting Prophage-Mediated Lysis for Biotherapeutic Release by *Lactobacillus reuteri*

**DOI:** 10.1128/AEM.02335-18

**Published:** 2019-05-02

**Authors:** Laura M. Alexander, Jee-Hwan Oh, Donald S. Stapleton, Kathryn L. Schueler, Mark P. Keller, Alan D. Attie, Jan-Peter van Pijkeren

**Affiliations:** a Department of Food Science, University of Wisconsin—Madison, Madison, Wisconsin, USA; b Department of Biochemistry, University of Wisconsin—Madison, Madison, Wisconsin, USA; University of Georgia

**Keywords:** *Lactobacillus reuteri*, bacteriophage, leptin, microbial delivery, probiotic, prophage, therapeutic

## Abstract

Lactobacillus reuteri has the potential to be developed as a microbial therapeutic delivery platform because of an established safety profile, health-promoting properties, and available genome editing tools. Here, we show that L. reuteri VPL1014 exhibits a low mutation rate compared to other Gram-positive bacteria, which we expect will contribute to the stability of genetically modified strains. VPL1014 encodes two biologically active prophages, which are induced during gastrointestinal transit. We hypothesized that intracellularly accumulated recombinant protein can be released following bacteriophage-mediated lysis. To test this, we engineered VPL1014 to accumulate leptin, our model protein, inside the cell. *In vitro* prophage induction of recombinant VPL1014 released leptin into the extracellular milieu, which corresponded to bacteriophage production. We also employed a plasmid system that does not require antibiotic in the growth medium for plasmid maintenance. Collectively, these data provide new avenues to exploit native prophages to deliver therapeutic molecules.

**IMPORTANCE** Lactic acid bacteria (LAB) have been explored as potential biotherapeutic vehicles for the past 20 years. To secrete a therapeutic in the extracellular milieu, one typically relies on the bacterial secretion pathway, i.e., the Sec pathway. Overexpression of a secreted protein can overload the secretory pathway and impact the organism’s fitness, and optimization of the signal peptide is also required to maximize the efficiency of the release of mature protein. Here, we describe a previously unexplored approach to release therapeutics from the probiotic Lactobacillus reuteri. We demonstrate that an intracellularly accumulated recombinant protein is released following prophage activation. Since we recently demonstrated that prophages are activated during gastrointestinal transit, we propose that this method will provide a straightforward and efficient approach to deliver therapeutics *in vivo*.

## Abstract (precis)

Lactic acid bacteria (LAB) have been explored as potential biotherapeutic vehicles for the past 20 years. To secrete a therapeutic in the extracellular milieu, one typically relies on the bacterial secretion pathway, i.e., the Sec pathway. Overexpression of a secreted protein can overload the secretory pathway and impact the organism’s fitness, and optimization of the signal peptide is also required to maximize the efficiency of the release of mature protein. Here, we describe a previously unexplored approach to release therapeutics from the probiotic Lactobacillus reuteri. We demonstrate that an intracellularly accumulated recombinant protein is released following prophage activation. Since we recently demonstrated that prophages are activated during gastrointestinal transit, we propose that this method will provide a straightforward and efficient approach to deliver therapeutics *in vivo*.

## INTRODUCTION

Lactic acid bacteria (LAB) are a diverse group of Gram-positive, non-spore-forming bacteria. Representative genera include *Lactococcus*, *Streptococcus*, *Lactobacillus*, *Leuconostoc*, and *Pediococcus*. Lactic acid is the main end product of glucose fermentation, which in homofermentative LAB yields two molecules of lactic acid per molecule of glucose, whereas heterofermentative LAB convert glucose to a mixture of carbon dioxide, ethanol, and lactic acid (1). LAB can be found in various food-related ecosystems, including plant materials and traditional fermented foods (e.g., kimchi), and are of interest to the food industry, as several LAB strains produce antimicrobial molecules, i.e., bacteriocins (2). Some of these bacteriocins are effective in killing foodborne pathogenic bacteria, including Listeria monocytogenes (3). Due to the long history of safe consumption, the U.S. Food and Drug Administration deemed many LAB strains to be generally recognized as safe (GRAS) (4). The safety of the probiotic itself, combined with the fact that several strains have health-promoting properties, have put LAB in the spotlight to be genetically modified as factories for the production of recombinant, therapeutic proteins.

Members of the genera *Lactococcus* and *Lactobacillus* are excellent hosts for the production of enzymes, biofuels, prophylactics, and therapeutics (5–8). Due to the ability of LAB to survive gastrointestinal (GI) transit and interact with mucosal environments, the oral or intranasal LAB-mediated delivery of vaccines and therapeutics is an attractive alternative to intravenous or intramuscular administration of antigenic molecules (9). Recombinant LAB have demonstrated efficacy in animal models for the delivery of vaccines to target Clostridium difficile (10), Helicobacter pylori (11–13), human papillomavirus (14–16), and influenza viruses (17–21). Lactococcus lactis, a species commonly found in milk products, has been extensively explored as a delivery vehicle and has been engineered to produce a variety of therapeutics, including interleukin-10 (IL-10) (22, 23), leptin (24), and the HIV-1 virucide cyanovirin protein (25). Phase I clinical trials with recombinant L. lactis secreting IL-10 demonstrated that the treatment was safe, but no significant decrease in disease activity in patients suffering from Crohn’s disease was observed (23). While further investigation is needed to better translate success in animal models to human applications, it is evident that L. lactis paved the way to develop LAB, including Lactobacillus reuteri, as therapeutic delivery vehicles.

Lactobacillus reuteri is a gut symbiont species found in the intestine of various vertebrates, including humans, pigs, cattle, rodents, sheep, and birds (26–31). The organism has evolved to thrive in the intestine, and select strains exhibit probiotic features, including modulation of inflammation (32–35), prevention of bone loss in menopausal females (36), and production of reuterin, an antimicrobial molecule that has activity against Escherichia coli O157:H7 and Listeria monocytogenes, for example (37, 38). Genetic tools, such as single-stranded DNA recombineering (39), CRISPR-Cas genome editing (40), and a counterselection marker (41), have been developed for L. reuteri and provide the species the potential to be developed as a therapeutic delivery vehicle.

To secrete therapeutic molecules from bacteria, research groups have exclusively exploited the secretory pathway. The secretory pathway is an export machinery responsible for transporting a variety of proteins into and across the plasma membrane of bacteria (42). For biotherapeutic delivery, a signal peptide targets the therapeutic protein for secretion and is recognized by signal peptidase I (SPaseI), a transmembrane protein that facilitates translocation of the therapeutic fusion protein across the bacterial cell membrane and that cleaves the signal peptide (43). The mature protein either remains associated with the cell, is anchored to the cell surface, or is released into the extracellular space (43). However, exploiting the secretory pathway to secrete high levels of recombinant protein can impose a burden on the cell. Additionally, the design of the fusion protein comes with several challenges. For example, the amino acid composition of the signal peptide combined with the N-terminal sequence of the mature protein is critical for the optimal processing of SPaseI (44). The SPaseI efficiency in E. coli, Bacillus subtilis, or select lactobacilli does not always extend to other lactobacilli, and the extraordinary genetic diversity of members of the genus *Lactobacillus* likely contributes to this (45–49). Clearly, passage of recombinant proteins through the secretory pathway can be a bottleneck to efficiently deliver content and, thus, requires optimization to maximize efficiency (43, 44).

Another important consideration in the engineering of bacteria as biotherapeutic delivery vehicles is finding alternatives to antibiotic selection for recombinant plasmid maintenance. Antibiotic alternatives should eliminate both the need for antibiotics in the growth medium and concerns about spreading antibiotic resistance genes to the host microbiota. Engineered auxotrophy provides an elegant solution to this problem. By modifying the bacterium for auxotrophy to an essential amino acid, for example, the relevant gene can then be supplied in *trans* on the desired plasmid expression system. Examples of this include the use of triosephosphate isomerase in E. coli (50), threonine auxotrophy in L. lactis (51), and thymidine synthase in Lactobacillus acidophilus (52).

In this study, we explored the potential of L. reuteri VPL1014 to be a therapeutic delivery platform. Rather than using the secretion pathway to secrete proteins into the environment, we exploited native prophages of L. reuteri to lyse the bacterium and to release our model protein, leptin. Finally, we employed a thymidine synthase-based plasmid system that can be stably maintained in the cell without the need for antibiotic selection.

## RESULTS

### L. reuteri VPL1014 has a low mutation rate and survives gastrointestinal transit in a mouse.

**FIG 1:**
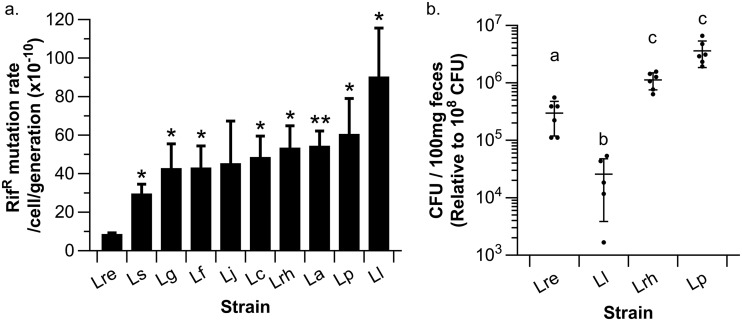
Assessment of potential biotherapeutic delivery vehicles. (a) Mutation rates of selected lactic acid bacteria determined by the FALCOR method (6). L. reuteri (Lre) exhibits a low mutation rate relative to other lactic acid bacteria, L. salivarius (Ls), L. gasseri (Lg), L. fermentum (Lf), L. jensenii (Lj), L. casei (Lc), L. rhamnosus (Lrh), L. acidophilus (La), L. plantarum (Lp), and L. lactis (Ll). *, *P* < 0.05; **, *P* < 0.01 (relative to L. reuteri). The results shown are averages from three independent experiments ± standard error. (b) LAB survival following GI transit in a mouse. L. reuteri [LR::*rpoB*(H488R)], L. rhamnosus, and L. plantarum survived GI transit at least 10-fold better than L. lactis (*P* < 0.001, Tukey’s HSD). Each dot represents a single mouse. Different letters indicate statistical differences between the respective treatment groups.

One of the long-term goals of our research group is to develop lactic acid bacteria (LAB) as a platform to deliver therapeutics in the gastrointestinal (GI) tract. Potential strains for this purpose ideally (i) have a low mutation rate to preserve genetic integrity, (ii) can survive GI transit, and (iii) are genetically accessible. First, we performed a mutation rate analysis on 10 select LAB, including Lactococcus lactis, to determine the number of mutations acquired per cell per generation. We observed that L. reuteri VPL1014 exhibited the lowest mutation rate (8.7 × 10^−10^ mutations/cell/generation), which was 9.4-fold lower than that of L. lactis (90.4 × 10^−10^ mutations/cell/generation) (Fig. 1a). The mutation rate of L. reuteri VPL1014 was 3.4-fold lower than that of the strain with the second-lowest mutation rate, Lactobacillus salivarius (29.7 × 10^−10^ mutations/cell/generation), with the mutation rate in the latter being comparable to the mutation rates of the seven remaining lactobacillus strains, ranging from 42.9 × 10^−10^ (Lactobacillus gasseri ATCC 33323) to 60.6 × 10^−10^ (Lactobacillus plantarum BAA-793) mutations/cell/generation. Thus, the mutation rate varies considerably within the genus *Lactobacillus*.

Next, we compared a subset of LAB—L. reuteri VPL1014, L. plantarum BAA-793, Lactobacillus rhamnosus ATCC 53103, and L. lactis NZ9000—for their ability to survive passage through the mouse GI tract. To identify and quantify the strains, we isolated rifampin-resistant derivatives either by selecting strains that have naturally acquired mutations to render rifampin resistance (L. rhamnosus, L. plantarum) or by mutating the *rpoB* gene to yield a rifampin-resistant phenotype, which we accomplished by single-stranded DNA recombineering [creating L. lactis LC::*rpoB*(H486N) and L. reuteri LR::*rpoB*(H488R)] (39). We administered the bacteria to mice (*n* = 6/group) for two consecutive days at 10^8^ CFU per day, and at 16 h after the final gavage we quantified the viable bacteria in the feces. L. rhamnosus and L. plantarum were the most robust in their ability to survive GI transit (10^6^ CFU/100 mg feces), while L. reuteri and L. lactis were recovered at 10^5^ and 10^4^ CFU/100 mg feces, respectively (Fig. 1b). L. lactis MG1363—the precursor strain of NZ9000—has successfully been developed as a therapeutic delivery vehicle in clinical trials (23). We concluded that the intermediate survival capacity of L. reuteri VPL1014 is not a limiting factor in the development of this strain as a therapeutic delivery vehicle. The combination of the low mutation rate, the ability to survive passage through the GI tract at levels that exceed those of the established L. lactis delivery vehicle, and the extended genome editing toolbox that has been developed for use in L. reuteri (single-stranded DNA recombineering, CRISPR-Cas genome editing, and a recently developed counterselection marker [39–41]) led us to select L. reuteri VPL1014 for development as a therapeutic delivery vehicle. To evaluate L. reuteri VPL1014 as a delivery vehicle, we chose the hormone leptin as our model molecule.

### Secreted leptin is inefficiently processed by Lactobacillus reuteri VPL1014.

**FIG 2:**
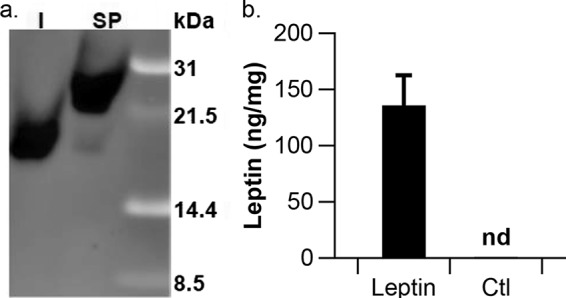
L. reuteri-mediated leptin production. (a) Western blotting results for intracellularly accumulated leptin indicated that leptin-3×FLAG is produced at the expected size, while the majority of secreted leptin is incorrectly cleaved. Lane I, LR/pLeptin-3×FLAG (19 kDa); lane SP, secreted leptin LR/pSP-Leptin-3×FLAG (23 kDa). (b) ELISA confirmed leptin production by LR/pLeptin. Ctl, LR/pCtl; nd, not detected. The results shown are averages from three independent experiments ± standard error, normalized per 1 mg of cell pellet dry weight.

Leptin is produced by adipose tissue and modulates appetite in humans and mice by signaling satiety in the brain (53, 54). First, we engineered L. reuteri VPL1014 to produce 3×FLAG-tagged murine leptin, codon optimized for expression in L. reuteri, from the multicopy plasmid pJP028 (LR/pSP-Leptin-3×FLAG). By Western blot analysis, we demonstrated that recombinant leptin was produced by LR/pSP-Leptin-3×FLAG. However, our results suggested that the signal peptide was not processed efficiently; the size of the majority of the recombinant protein corresponded to that of the unprocessed precursor, while a small fraction of protein yielded the expected size for mature leptin (Fig. 2a). These findings were substantiated after we engineered L. reuteri to express leptin lacking a signal peptide (LR/pLeptin-3×FLAG): Western blot analysis demonstrated that the 3×FLAG-tagged leptin was produced at the expected size (19 kDa; Fig. 2a). To circumvent the use of a signal peptide to release the recombinant protein, we decided to pursue the development of L. reuteri to accumulate leptin within the cell for subsequent delivery.

First, we determined to what extent recombinant protein can be accumulated in the cells. To quantify the amount of leptin that was accumulated intracellularly, we lysed a ∼16-h culture of LR/pLeptin-3×FLAG by bead beating and subjected the cell-free supernatant to enzyme-linked immunosorbent assay (ELISA). Our results showed that approximately 132 ± 27.8 ng of leptin per 1 mg of cells (by cell dry weight) was produced by recombinant L. reuteri (Fig. 2b). As expected, we did not detect leptin in the lysate derived from L. reuteri VPL1014 harboring the pJP028 vector control plasmid (pCtl). Once we had determined that leptin could be accumulated to nanogram-per-milligram concentrations in culture medium, there was an opportunity to release recombinant protein following cell lysis. This approach would alleviate the concern of inefficient secretion and/or processing of the signal peptide and relieve the pressure on the bacterial secretion system to secrete recombinant protein. Therefore, we explored the use of bacteriophages to release recombinant protein from L. reuteri.

### Exploiting prophages to release the therapeutic molecule.

We recently demonstrated that L. reuteri ATCC PTA 6475, a precursor of VPL1014, encodes two biologically active prophages, which are bacterial viruses that are integrated in the bacterial genome (55). During GI transit, the prophages are activated, leading to the production of bacteriophages and an approximately 8-fold reduction in L. reuteri survival (55). We reasoned that we could exploit bacteriophage-mediated lysis to release a therapeutic molecule. To establish a proof of concept, we used an untagged version of the same construct described above (LR/pLeptin) and performed a mitomycin C induction experiment. Mitomycin C is a DNA-damaging agent that induces the SOS response of bacteria, which in turn activates lysogenic phage to lead to phage-mediated cell lysis (56, 57). LR/pLeptin and the wild-type strain harboring an empty vector control (LR/pCtl) were induced with mitomycin C (0.5 μg/ml) at an optical density at 600 nm (OD_600_) of 0.3. Before induction (time zero [*T*_0_]) and at 5 h after MitC induction (*T*_5_), we harvested culture supernatants to quantify leptin. We chose *T*_5_ as our endpoint in the analysis as no further reduction in optical density was observed compared to that at 6 h postinduction (*T*_6_) and beyond. At *T*_5_, we recovered 18.8-fold more leptin in the supernatant of the induced culture than in that of the uninduced control culture (51.6 ng/ml for the induced culture versus 2.6 ng/ml for the uninduced culture; *P* < 0.001). This suggests that prophage activation promotes the release of intracellularly accumulated protein from LR/pLeptin.

**FIG 3:**
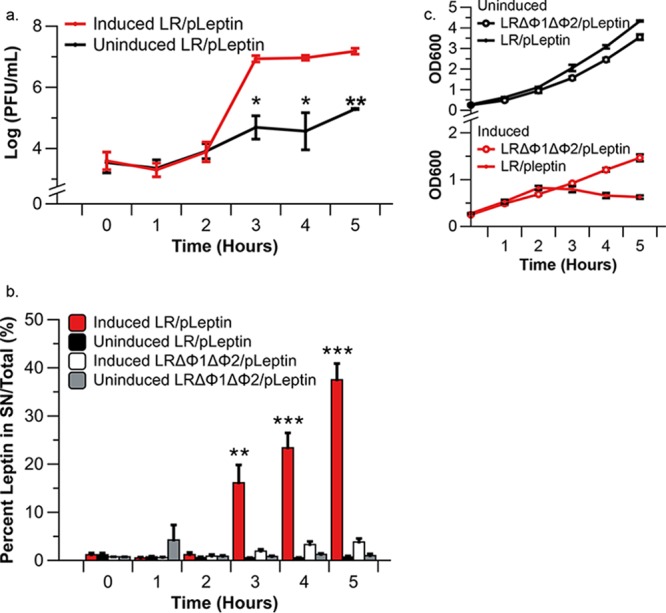
Leptin release from recombinant VPL1014 following mitomycin C treatment. (a) Numbers of PFU derived from leptin-producing VPL1014 culture. No PFU were produced by induced or uninduced LRΔΦ1ΔΦ2/pLeptin. The results shown are averages from three independent experiments ± standard error. (b) ELISA data showing the percentage of total leptin (from the supernatant [SN] plus the cell lysate) released into the extracellular milieu. The results shown are averages from three independent experiments ± standard error. (c) Growth curves of uninduced LR/pLeptin, uninduced LRΔΦ1ΔΦ2/pLeptin, induced LR/pLeptin, and induced LRΔΦ1ΔΦ2/pLeptin are shown. Asterisks indicate statistical differences between respective induced and uninduced groups. *, *P* < 0.05; **, *P* < 0.01; ***, *P* < 0.001 (Tukey’s HSD). The results shown are averages of three independent experiments ± standard error.

Next, we examined the dynamics of phage-induced lysis and leptin release. We induced LR/pLeptin with mitomycin C and tracked the number of PFU per milliliter, leptin release, and growth (OD_600_) every hour postinduction. We report leptin release as the percentage of leptin detected in the supernatant compared to the total amount of leptin present in the supernatant and cells. As expected, induction of LR/pLeptin resulted in an increase in the amount of PFU over time (Fig. 3a). Compared to the amount at 2 h postinduction (*T*_2_), at 3 h postinduction (*T*_3_) we observed an exponential increase in the number of PFU per milliliter (3.8 log PFU/ml at *T*_2_ versus 6.9 log PFU/ml at *T*_3_; *P* < 0.02; Fig. 3a), which corresponded to a significant increase in the percentage of leptin released (1.21% at *T*_2_ versus 16.1% at *T*_3_; *P* < 0.05; Fig. 3b). At the following time points, *T*_4_ and *T*_5_, the cell density was reduced (OD_600_ = 0.79 at *T*_3_ versus 0.66 *T*_4_ and 0.63 at *T*_5_), signifying cell lysis (Fig. 3c). At 5 h postinduction, 38% of the leptin was released into the culture supernatant. In the uninduced LR/pLeptin control, the number of PFU increased slightly at 3 h postinduction (3.9 log PFU/ml at *T*_2_ versus 4.7 log PFU/ml at *T*_3_; *P* < 0.05), while the percentage of released leptin remained steady (0.62% at *T*_2_ versus 0.58% at *T*_3_; *P* > 0.05) (Fig. 3). Together, these data suggest that bacteriophage-mediated lysis contributes to leptin release. To further substantiate this, we expressed leptin in LRΔΦ1ΔΦ2, a derivative which lacks prophages (55). Mitomycin C induction of the LRΔΦ1ΔΦ2/pLeptin culture did not induce lysis, and leptin release was marginal, as we recovered only 1.9% and 3.82% leptin at *T*_3_ and *T*_5_, respectively (Fig. 3). Collectively, we demonstrated that an exponential increase in phage production releases recombinant protein into the extracellular milieu, which provides a novel approach to deliver therapeutics. However, LR/pLeptin requires further optimization prior to *in vivo* studies. For example, as of now, we require antibiotics to maintain the recombinant plasmid in the cell. To overcome this, we focused next on an approach to eliminate the need for antibiotics in the growth medium.

### pMutL-ThyA stably maintains LR/pLeptin in the absence of antibiotic selection.

**FIG 4:**
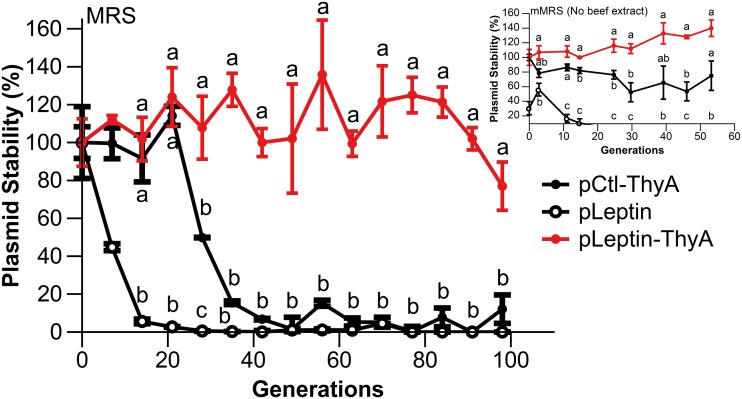
Plasmid stability of the pLeptin-ThyA construct in LRΔ*thyA*::*rpoB*(H488R). Plasmid stability is represented by the percentage of cells from plain MRS broth that retained pLeptin-ThyA, pCtl-ThyA, or pLeptin over the course of ∼100 generations without antibiotic in the medium. (Inset) Plasmid stability of pLeptin-ThyA, pCtl-ThyA, or pLeptin from mMRS without thymidine (no beef extract) (*P* < 0.01, Tukey’s HSD). The results shown are averages from three independent experiments ± standard error. Different letters indicate statistical differences between the respective treatment groups.

The gene *thyA* encodes thymidylate synthase, which converts dUMP to dTMP, also known as thymidine (52). Previously, it was demonstrated that plasmid expression of *thyA* in a genetic background that lacks *thyA* renders stable plasmid maintenance (52, 58, 59). To establish a proof of concept in L. reuteri, we first inactivated *thyA* by single-stranded DNA recombineering and confirmed that the resultant strain was auxotrophic for thymidine (data not shown). Next, we modified the leptin expression vector, which contains gene cassettes that encode resistance to chloramphenicol (Cm) and erythromycin (Em). We replaced the gene cassette encoding chloramphenicol resistance with *thyA* and transformed the resultant construct into LRΔ*thyA*::*rpoB*(H488R) to yield LRΔ*thyA*::*rpoB*(H488R)/pLeptin-ThyA. For this experiment, we purposely maintained the marker encoding erythromycin resistance, which allowed us to accurately determine plasmid stability within the population. As controls, we included an empty vector control (pCtl-ThyA) and the vector encoding leptin lacking *thyA* (pLeptin). To determine plasmid stability, we passaged the strains for ∼100 generations in De Man, Rogosa, and Sharpe (MRS) medium without antibiotic selection, after which we determined the total number of CFU and the number of CFU which were resistant to erythromycin. After ∼100 generations, we confirmed that 12% ± 7.5% of the cells retained the empty vector control (pCtl-ThyA), while we did not recover any cells that retained the plasmid expressing leptin (pLeptin), whereas the plasmid expressing ThyA (pLeptin-ThyA) was present in 77% ± 12% of the cells in the population (Fig. 4). We also performed this experiment in modified MRS medium (mMRS) without antibiotic or thymidine and observed that after 50 generations, pCtl-ThyA and pLeptin-ThyA were retained at 62% ± 20% and 100% ± 11%, respectively, while pLeptin was lost after 25 generations (Fig. 4, inset). Thus, the combination of LRΔ*thyA*::*rpoB*(H488R) and in *trans* expression of ThyA increased plasmid stability compared to that in the controls. In conclusion, LRΔ*thyA:rpoB*(H488R)/pLeptin-ThyA now constitutes a strain that does not require antibiotic in the growth medium for plasmid retention, which we will exploit in future *in vivo* studies.

## DISCUSSION

In this study, we explored the novel approach of exploiting the L. reuteri VPL1014 prophage for the release of an intracellularly accumulated biotherapeutic. Based on its genetic stability, *in vivo* survivability, and genetic accessibility, we pursued L. reuteri VPL1014 as a delivery platform. After engineering L. reuteri VPL1014 to produce leptin within the cell, we demonstrated that we can exploit phage-mediated lysis to release leptin, while we also developed a plasmid system that does not require antibiotic selection.

An important criterion for the selection of L. reuteri VPL1014 for development as a biotherapeutic delivery platform was the low mutation rate of this strain. The underlying mechanisms that explain our findings remain speculative, but it is possible that differences in the activity of MutS, a conserved protein that repairs mismatches that occur during replication (60, 61), could contribute to the differences in the mutation rates of lactobacilli. We determined that L. reuteri ATCC PTA 6475—a precursor of VPL1014—and DSM20016^T^ have similar mutation rates (data not shown), and our finding could provide a potential explanation of the clonal nature of human-derived L. reuteri strains ATCC PTA 6475, JCM1112, and DSM20016^T^, which were isolated from different continents yet which have nearly identical genomes (62). From a practical standpoint, our finding is interesting because the low mutation rate of L. reuteri VPL1014 results in a recombinant strain that acquires few single-nucleotide polymorphisms (SNPs) compared to other LAB strains, including L. lactis, which has a mutation rate that is nearly 10-fold higher than that of L. reuteri. Thus, we expect that the low mutation rate of L. reuteri VPL1014 contributes to the genetic stability of our engineered strains.

Although the mutation rate of L. reuteri VPL1014 was one of the main criteria for selection of this strain for further development as a therapeutic, we observed that the ability to survive gastrointestinal transit was intermediate compared to that of the other strains tested. In fact, L. plantarum BAA-793 and L. rhamnosus GG survived GI transit better than L. reuteri VPL1014. Logically, differences in the ability to survive exposure to acids, i.e., stomach and bile, may explain our observation. In addition, differences in prophage activation during GI transit could contribute to different efficiencies of survival of GI transit. Recently, we demonstrated that prophages in L. reuteri are activated during GI transit. A mutant lacking the prophage genomes was recovered at approximately 8-fold higher levels than L. reuteri isolates harboring prophages (55). At this point, we cannot compare our recent findings to those in the literature, as studies pertaining to prophage activation of gut symbionts during GI transit are in their infancy. However, the genomes of both L. plantarum and L. rhamnosus encode biologically active prophages, as demonstrated by mitomycin C induction experiments (data not shown), which means that prophages cannot be excluded as a factor contributing to differences in GI survival. Less surprising was our finding that L. lactis was the least robust during GI transit. L. lactis is commonly found in milk products and has not evolved to thrive in the intestinal environment, unlike L. reuteri, for example (63, 64). Although L. lactis NZ9000 does not carry biologically active prophages that can contribute to reduced GI survival (65), it has been shown that L. lactis is more sensitive to bile acids (66), which could partially explain the organism’s reduced ability to survive GI transit.

Induction of prophages in L. reuteri during GI transit, thereby causing cell lysis in the gut and the release of intracellular contents, can be used to deliver therapeutics. Recently published findings showed that the intracellular accumulation of interleukin-22 (IL-22) in L. reuteri leads to the release of IL-22 during gastrointestinal transit, as demonstrated by the induced expression of *regIIIγ*, a gene regulated by IL-22 (67). With a daily dosage of 10^7^ CFU of recombinant L. reuteri for 7 days, Hendrikx et al. observed a decrease in liver damage in a model of murine alcohol-induced liver disease (67). Despite this exciting result, we do not currently have evidence that this method would result in the systemic delivery of a therapeutic, which would be necessary for leptin to acquire biological efficacy. Achieving systemic delivery may also depend on the properties of the biotherapeutic molecule itself, such as polarity and hydrophobicity. For any potential therapeutics, individual *in vivo* studies must be conducted to demonstrate delivery and efficacy.

There may be multiple advantages to using phage-mediated lysis as an approach. First, we have shown that diet can alter bacteriophage production in L. reuteri (55), which opens up the exciting opportunity to explore diet as a means to control the release of therapeutics. Second, prophage-mediated delivery of therapeutics could reduce the total number of viable recombinant bacteria, thereby contributing to biological containment. However, studies to maximize biological containment to fully eradicate recombinant L. reuteri remain needed: although L. reuteri has proven to be safe for consumption (68–74), strategies need to be in place to halt the release of recombinant protein to better control both dosage and a possible undesirable side effect. Third, using bacteriophages to release therapeutic molecules alleviates the need to use the native secretion system to deliver a protein. This means that there is no need to identify the optimal signal peptide for secretion, which is known to be dependent on the sequence of the mature protein and the screening for which can be a time-consuming process (44). Also, we expect that we are imposing a lower burden on cell metabolism.

In future studies, we plan to address the biological containment of our genetically modified organism. We will insert the leptin gene into the L. reuteri chromosome under the control of the EF-Tu promoter and maximize the level of phage-mediated lysis by hijacking the phage regulatory proteins. We expect that this will result in a food-grade, antibiotic-free, biologically contained delivery platform. At a minimum, the concept of engineering L. reuteri for the intracellular accumulation of a product combined with phage-mediated lysis can be applied to studies to explore the local effect of molecules that would otherwise be difficult to deliver to the GI tract. The exploitation and study of probiotic prophages can both result in an effective, biologically contained therapeutic delivery vehicle and provide further insight into the role of phages in probiotic efficacy. In conclusion, our work presents a novel method to accomplish the delivery of therapeutics by exploiting phage-mediated lysis for therapeutic release by L. reuteri VPL1014.

## MATERIALS AND METHODS

### Bacterial strains and media.

The bacterial strains and plasmids used in this study are listed in Table 1. Escherichia coli EC1000 was used as an intermediate cloning host and was cultured at 37°C in lysogeny broth (LB; Teknova). Competent cells of E. coli EC1000 were prepared as described previously (75). Lactobacillus reuteri isolates lacking prophages, LRΔΦ1ΔΦ2 (VPL4121) and LRΔΦ1ΔΦ2Δ*attB1ΔattB2* (VPL4090, lytic host) were constructed previously (55). Construction of the rifampin-resistant strains used in the *in vivo* survival experiment was achieved through single-stranded DNA recombineering that targeted the *rpoB* gene [L. lactis (VPL4005) and LR::*rpoB*(H488R) (VPL4126)] as described previously (39) or by random mutant isolation (L. rhamnosus VPL4141 and L. plantarum VPL4142). Lactobacilli were grown in De Man, Rogosa, and Sharpe (MRS) medium (Difco, BD Biosciences) under hypoxic conditions (5% CO_2_, 2% O_2_) on agar plates at 37°C or in broth (static) at 37°C in a conventional aerated incubator. L. reuteri competent cells were prepared as described previously (40). L. lactis was grown in M17 broth (Difco) supplemented with 0.5% (wt/vol) glucose at 30°C. As needed, erythromycin was supplemented at 5 μg/ml for the L. reuteri strains and 300 μg/ml for E. coli EC1000. Chloramphenicol was added as needed at 5 μg/ml or 20 μg/ml for L. reuteri and E. coli EC1000, respectively. Rifampin was added as needed at 25 μg/ml. To select for LRΔ*thyA*::*rpoB*(H488R) (VPL4143), we used modified MRS medium lacking beef extract (mMRS-BE), which has the following ingredients: peptone (10 g/liter; BD Biosciences), yeast extract (5 g/liter; IBI Scientific), Tween 80 (1 ml/liter; Sigma-Aldrich), ammonium citrate dibasic (2 g/liter; Sigma-Aldrich), dipotassium phosphate (2 g/liter; Fisher Scientific), sodium acetate (1 g/liter; Sigma-Aldrich), magnesium sulfate (0.1 g/liter; Fisher Scientific), manganese sulfate (0.05 g/liter; Sigma-Aldrich), and glucose (100 mM; Sigma-Aldrich). mMRS-BE was supplemented with trimethoprim (40 μg/ml) and/or thymidine (50 μg/ml) as needed.

**TABLE 1:** Bacterial strains and plasmids used in this study

Strain or plasmid^*a*^	Characteristics^*b*^	Source or reference^*c*^
Strains		
E. coli EC1000	Derivative of E. coli MC1000 in which *repA* is integrated in the chromosome	85
L. reuteri		
VPL1014	Human breast milk isolate	Lab stock
VPL4090	Mutant lacking both active phages and *attB* sites, lytic host	55
VPL4121	Mutant lacking both active phages, restored *attB* sites, LRΔΦ1ΔΦ2	55
VPL4224	Rif^r^ mutant generated with oVPL236 for mutation in *rpoB* gene (H488R)	55
VPL4243	Derived from VPL4224, mutations introduced in *thyA* (Y38*, Q39S, M40L) with oVPL1670	This work
L. rhamnosus		
ATCC 53103	Human fecal isolate	ATCC
VPL4141	Rif^r^ natural mutant isolated from MRS medium-Rif25 plate	This work
L. casei BFLM218	Human fecal isolate	35
L. fermentum ATCC 14931	Fermented beet isolate	ATCC
L. plantarum		
ATCC BAA-793	Human saliva isolate	ATCC
VPL4142	Rif^r^ natural mutant isolated from MRS medium-Rif25 plate	This work
L. salivarius CCUG 47825	Human blood isolate	CCUG (86)
L. gasseri ATCC 33323	Human intestinal isolate	ATCC
Lactococcus lactis subsp. *cremoris*		
NZ9000	Dairy starter, derivative of MG1363, *pepN*::*nisRK*	87
VPL4005	Rif^r^ mutant generated with oVPL234 for mutation in *rpoB* gene (H486N)	This work
L. jensenii ATCC 25258	Human vaginal isolate	ATCC
L. acidophilus ATCC 4356	Human isolate	ATCC
Plasmids		
pVPL2042	Em^r^, pNZ8048 derivative; the Cm^r^ marker was replaced by an Em^r^ marker	Lab stock
pVPL3583	pJP028 vector control (pCtl)	This work
pVPL3585	pJP028 derivative, pNZ-EFTu-SP-Leptin	This work
pVPL3752	pJP028 derivative, pJP-EFTu-SP-Leptin-3×FLAG	This work
pVPL3791	pJP028 derivative lacking the signal peptide, pNZ-EFTu-Leptin	This work
pVPL3795	pJP028 derivative, pJP-EFTu-Leptin-3×FLAG	This work
pVPL31131	pJP028 derivative, pJP-EFTu-Leptin-ThyA	This work
pVPL31134	pJP028 derivative, pCtl-ThyA	This work

a VPL, van Pijkeren Laboratory strain identification number; pVPL, van Pijkeren Laboratory plasmid identification number.

b *repA*, gene for replication initiation protein; *attB*, phage insertion site; Em^r^, erythromycin resistant; Cm^r^, chloramphenicol resistant; Rif^r^, rifampin resistant; Rif25, rifampin at 25 μg/ml; *rpoB*, gene encoding β subunit of RNA polymerase, homolog of LAR_1402 in L. reuteri JCM1112; *thyA*, gene encoding thymidylate synthase, homolog of LAR_0739 in L. reuteri JCM1112. An asterisk (*) indicates a stop codon. The locus tags listed can be found at https://www.ncbi.nlm.nih.gov.

c ATCC, American Type Culture Collection.

### Mutation rate analysis.

Bacterial cultures were incubated for 16 h, subcultured to 10^3^ CFU/ml, and subsequently split into 24 wells (1 ml/well) in a deep-well 96-well plate (Celltreat). Following 48 h of incubation, the total viability and the total number of rifampin-resistant cells were determined by spread plating. The mutation rate was calculated with the fluctuation analysis calculator (FALCOR) using the Ma-Sandri-Sarkar maximum likelihood estimator (MSS-MLE), as described previously (76).

### Ethics statement.

All mouse experiments were performed in accordance with NIH guidelines, Animal Welfare Act, and U.S. federal law and were approved by the Application Review for Research Oversight at Wisconsin (ARROW) committee and overseen by the Institutional Animal Care and USE Committee (IACUC) under protocol ID A005821-A03. All mice were housed in an animal research facility (Biochemistry B145) at the University of Wisconsin accredited by the Association of Assessment and Accreditation of Laboratory Animal Care (AAALAC) International.

### Bacterial survival following gastrointestinal transit.

Twenty-four 6-week-old male C57BL/6J mice were purchased from The Jackson Laboratory (Bar Harbor, ME). Prior to the start of the experiment, the animals were allowed to adjust to the new environment for 1 week. The animals were individually housed in an environmentally controlled facility with a 12-h light and 12-h dark cycle. Food (standard chow; LabDiet, St. Louis, MO) and water were provided *ad libitum*. Mice (*n* = 6/group) were gavaged for two consecutive days with 100 μl a phosphate-buffered saline (PBS) suspension containing 10^9^ CFU/ml of rifampin-resistant L. reuteri ATCC PTA 6475, L. lactis NZ9000, L. rhamnosus GG, or L. plantarum BAA-793. Fecal samples were collected from the bedding at 16 h after the last oral administration and weighed. The fecal material was resuspended in PBS to 100 mg/ml and plated on MRS agar plates (or GM17 for L. lactis) containing 25 μg/ml rifampin. Cell viability counts were normalized per 10^8^ CFU.

### Heterologous expression of leptin: LR/pLeptin.

All oligonucleotides are listed in Table 2. To construct LR/pLeptin, we amplified the backbone of pJP028 (derived from pNZ8048) with primer pair oVPL1200-oVPL1286, followed by DpnI treatment (Thermo Scientific). The sequence encoding murine leptin was obtained from NCBI (GenBank accession number ADM72802.1). We codon optimized the leptin sequence for expression in L. reuteri with the OPTIMIZER web server (77, 78), followed by synthesis with gBlock gene fragments (Integrated DNA Technologies) (Table 2) and amplification with oligonucleotide pair oVPL1348-oVPL1349. All amplicons were purified (GeneJET PCR purification kit; Thermo Fisher), quantified (Qubit fluorometric quantification; Life Technologies), and phosphorylated (T4 polynucleotide kinase; Thermo Fisher) and then subjected to the ligation cycle reaction (LCR), as described previously (79). To clone leptin, we used bridging oligonucleotides oVPL1350 and oVPL1351. The resulting LCR mixture was transformed into E. coli EC1000 (VPL3481), and cloning of the leptin gene into pJP028 was confirmed by PCR (*Taq* polymerase; Denville Scientific) using oligonucleotides oVPL329 and oVPL363. The resultant construct was named pNZ-SP-Leptin.

**TABLE 2:** Oligonucleotides used in this study

Oligonucleotide name^*a*^	Sequence (5′–3′)^*b*^	Target/comment (reference)^*c*^
oVPL234	GAGATACCACCAGGTCCTAAGGCAGAGAAACGACGTTTGTTGCTAAGCTCAGACAAAGGATTATGTTGGTCCATAAATTGT	Recombineering oligonucleotide for L. lactis *rpoB* mutant
oVPL236	TCAAACCACCAGGACCAAGCGCTGAAAGACGACGCTTTCTGCTTAATTCACCTAATGGGTTGGTTTGATCCATGAACTGG	Recombineering oligonucleotide for L. reuteri *rpoB* mutant
oVPL329	ATTCCTTGGACTTCATTTACTGGGTTTAAC	Rev, for pJP028 insertion screening
oVPL362	TTGATATGCCTCCTAAATTTTTATCTAAAG	Rev, for pJP028 insertion screening
oVPL363	TAATATGAGATAATGCCGACTGTAC	Fwd, for pJP028 insertion screening
oVPL736	TGAATGAGTGAGTCAACTTG	Fwd, amplified pMutL of L. reuteri
oVPL1199	ATGTTATCGAAGAATAATCGAAAGG	Fwd, amplified signal peptide on pJP028
oVPL1200	TGTATTAGCAGAAGCATTCATCC	Rev, amplified signal peptide on pJP028
oVPL1286	TGATCTTTGAACCAAAATTAG	Fwd, amplified pJP028 backbone
oVPL1348	GTTCCAATTCAAAAAGTTCAAGATG	Fwd, amplified murine leptin from gBlock gene fragments, codon optimized for L. reuteri (77, 78)
oVPL1349	ACATTCTGGACTAACATCTAATTG	Rev, amplified murine leptin from gBlock gene fragments, codon optimized for L. reuteri (77, 78)
oVPL1350	TTCATGGGGATGAATGCTTCTGCTAATACAGTTCCAATTCAAAAAGTTCAAGATGATACT	LCR bridging oligonucleotide for leptin insertion for pJP028:SP:leptin
oVPL1351	TTACAACAATTAGATGTTAGTCCAGAATGTTGATCTTTGAACCAAAATTAGAAAACCAAG	LCR bridging oligonucleotide for leptin insertion for pJP028:SP:leptin
oVPL1408	AGAAAACCGACTGTAAAAAGTACAG	Rev, amplified backbone of pJP028 for promoter swap
oVPL1409	AGAAAACCGACTGTAAAAAGTACAGTCGGCTGAATGAGTGAGTCAACTTGAATTATTTGC	LCR bridging oligonucleotide for swapping in EF-Tu promoter in pJP028
oVPL1410	GCAGCAGAAATTGAAATAAGGTGATATTTAATGTTATCGAAGAATAATCGAAAGGAACAA	LCR bridging oligonucleotide for swapping in EF-Tu promoter in pJP028
oVPL1447	CGAATTAATAGAAAAACATTAGTCAAATAC	Fwd, amplified EF-Tu promoter
oVPL1448	TAATGAAAACCTCCTGATAATTTACAAG	Rev, amplified EF-Tu promoter
oVPL1670	CGTTAAAATAGGAAAACCTTTGCTTAGGTCAAATCGCA**AGCTT**TATCCGAAAACAGATTTAGTACCTGTTCCTGTCCGAT	Recombineering oligonucleotide for the Δ*thyA* mutant, introducing Y38*, Q39S, and M40L; bold nucleotides introduce 5 adjacent mismatches to the wild-type sequence
oVPL1671	GCTATTTCTTAGATAAAGTGGCTGAC	Fwd, for screening of the Δ*thyA* mutant (Y38*, Q39S, and M40L)
oVPL1672	TTTGCTTAGGTCAAATCGCAAGCTT	Rev, for screening of the Δ*thyA* mutant (Y38*, Q39S, and M40L)
oVPL1673	AAAATTGGAACATGGTGTGACATGGA	Rev, for screening of the Δ*thyA* mutant (Y38*, Q39S, and M40L)
oVPl1725	TTAAACTGCTACGGGAGCCTTG	Rev, amplified pMutL-ThyA
oVPL1810	**ATG**GTTCCAATTCAAAAAGTTCA	Fwd, amplified leptin and added ATG start codon (bold)
oVPL2112	**ATCTTTATAATCACCATCGTGATCTTTATAATC**ACATTCTGGACTAACATCTAATTG	Rev, amplified leptin and added 3×FLAG tag (bold) to CTD of leptin
oVPL2113	**CACGATATTGATTATAAAGATGATGATGATAAA**TGATCTTTGAACCAAAATTAG	Fwd, amplified pJP028 backbone and added 3×FLAG (bold) to CTD of leptin
oVPL2351	TAATCTCGCTTTGATTGTTCTATCG	Rev, amplified pJP028 backbone omitting Cm^r^ cassette
oVPL2352	AAGGAAGATAAATCCCATAAGGGCG	Fwd, amplified pJP028 backbone omitting Cm^r^ cassette

a oVPL, van Pijkeren Laboratory primer identification number.

b Boldface nucleotides were added to the primer (for example, as a start codon, stop codon, FLAG tag).

c Fwd, forward primer; Rev, reverse primer; *rpoB*, β subunit of RNA polymerase, homolog of LAR_1402 in L. reuteri JCM1112; *thyA*, thymidylate synthase, homolog of LAR_0739 in L. reuteri JCM1112; LCR, ligation cycle reaction; CTD, C-terminal domain; Cm^r^, chloramphenicol resistant. An asterisk (*) indicates a stop codon. The locus tags listed can be found at https://www.ncbi.nlm.nih.gov.

The purified pNZ-SP-Leptin plasmid was amplified with primer pair oVPL1199-oVPL1408 and was used as a template to construct pEFTu-SP-Leptin. A native constitutive promoter, EF-Tu, was amplified with primers oVPL1447 and oVPL1448 from pJG001 (a gift from Robert Britton) (41). Amplicons were subjected to LCR as described above with bridging oligonucleotides oVPL1409 and oVPL1410. The resulting plasmid, pNZ-EFTu-SP-Leptin, hereafter called pSP-Leptin, was transformed into L. reuteri VPL1014, resulting in LR/pSP-Leptin (VPL3585).

To generate a derivative lacking the signal peptide (pLeptin), we amplified the backbone of pSP-Leptin with oVPL1810 and oVPL1448, oligonucleotides that are located directly upstream and downstream of the sequence coding for the signal peptide, respectively. The resulting amplicon was fused by blunt-end ligation (T4 DNA ligase; Fisher Scientific) and transformed into L. reuteri VPL1014 and LRΔΦ1ΔΦ2 to yield LR/pLeptin (VPL3791) and LRΔΦ1ΔΦ2/pLeptin (VPL31067), respectively. The control plasmid was prepared by amplifying the backbone of pSP-Leptin with oVPL1408 and oVPL1286, oligonucleotides that are located directly upstream of the signal peptide and downstream of the sequence coding for leptin, respectively, followed by self-ligation and transformation into L. reuteri to yield LR/pCtl (VPL3583).

For Western blot analysis purposes, we inserted the sequence encoding a 3×FLAG tag to the 3′ proximal end of the leptin gene in plasmids pSP-Leptin and pLeptin. To accomplish this, we performed PCR using oligonucleotides oVPL2112 and oVPL2113, which are located on the 3′ end of the leptin gene and just downstream of leptin on the plasmid backbone, respectively. A tag of 66 bp was included on the 5′ end of each primer, which, following self-ligation, resulted in the DNA sequence coding for a 3×FLAG tag. The resulting plasmids were named pSP-Leptin-3×FLAG and pLeptin-3×FLAG, respectively, and established in L. reuteri VPL1014 to yield LR/pSP-Leptin-3×FLAG (VPL3752) and LR/pLeptin-3×FLAG (VPL3795), respectively.

### Construction of LRΔ*thyA*.

We inactivated *thyA* in a rifampin-resistant derivative of L. reuteri VPL1014 [LR::*rpoB*(H488R)] by single-stranded DNA recombineering as described previously (39). We previously engineered L. reuteri VPL1014 to be rifampin resistant to assess survival following GI transit [LR::*rpoB*(H488R) (VPL4126)] (55). LR::*rpoB*(H488R) expressing RecT was transformed with 100 μg of oVPL1670 to generate an in-frame stop codon in *thyA*, using methods described previously (39). To identify cells in which *thyA* was inactivated, we used positive selection with trimethoprim. Trimethoprim is toxic to cells producing ThyA because it prevents the reduction of the by-product dihydrofolate to tetrahydrofolate, thus inhibiting bacterial DNA synthesis (80). The selection medium was also supplemented with thymidine to allow the Δ*thyA* mutants to grow. Therefore, the LRΔ*thyA*::*rpoB*(H488R) mutants were selected by plating serial dilutions onto mMRS-BE supplemented with trimethoprim (40 μg/ml) and thymidine (50 μg/ml). The genotype of the colonies was confirmed by a mismatch amplification mutation assay (MAMA) PCR (81, 82) with oVPL1671, oVPL1672, and oVPL1673, followed by Sanger sequencing.

### Construction of pLeptin-ThyA.

To develop an expression vector without the need for antibiotic in the growth medium, the gene conferring chloramphenicol resistance in the pJP028 backbone was replaced with *thyA* (52). We amplified pLeptin with primers oVPL2351 and oVPL2352 to generate a plasmid backbone lacking the chloramphenicol resistance gene. To complement *thyA* in L. reuteri lacking *thyA*, we then put the *thyA* gene under the control of the L. reuteri pMutL promoter, a promoter located upstream of the gene encoding MutL, which is involved in DNA repair (83). We amplified pMutL:ThyA with oVPL736 and oVPL1725 using pSIP411:pMutL-ThyA as the template, which we subsequently fused by blunt-end ligation (T4 DNA ligase) to the pLeptin backbone to generate pLeptin-ThyA. The resulting construct, pLeptin-ThyA, was transformed into E. coli EC1000 to yield VPL31131. The purified pLeptin-ThyA plasmid was transformed into LRΔ*thyA*::*rpoB*(H488R), resulting in LRΔ*thyA*::*rpoB*(H488R)/pLeptin-ThyA (VPL31133). Transformants were selected on mMRS-BE agar harboring 5 μg/ml erythromycin (Em 5). LRΔ*thyA*::*rpoB*(H488R)/pLeptin-ThyA was then used for a plasmid stability experiment. A backbone control vector was prepared by amplifying pLeptin-ThyA, omitting leptin with oVPL1408-oVPL1286. This amplicon was then treated as described above and self-ligated with T4 ligase before transformation into LRΔ*thyA*::*rpoB*(H488R), resulting in LRΔ*thyA*::*rpoB*(H488R)/pCtl-ThyA (VPL31134). For comparison in the plasmid stability experiment, pLeptin lacking *thyA* was transformed into LRΔ*thyA*::*rpoB*(H488R), resulting in LRΔ*thyA*::*rpoB*(H488R)/pLeptin (VPL31135).

### Protein preparation, ELISA, and Western blotting.

**(i) Western blotting.** Intracellularly accumulated leptin from LR/pLeptin-3×FLAG and secreted leptin from LR/pSP-Leptin-3×FLAG were analyzed by Western blotting. Protein samples were prepared from ∼16-h cultures. LR/pLeptin-3×FLAG cells were harvested by centrifuging 1.5 ml of culture (1 min at 21,130 × *g*), the cell dry weight was measured for normalization purposes, and the cell pellet was washed once in 1.5 ml distilled water and resuspended in 500 μl lysis buffer (50 mM Tris HCl, pH 7.4, 150 mM NaCl, 1 mM EDTA, 1% Triton X-100). Approximately 100 μl of zirconia glass beads (BioSpec) was added to the suspension. Cells were vortexed six times for 30 s each time with 30-s intervals on ice. Lysates were harvested by transferring the supernatants into a fresh tube, adding 1 ml of lysis buffer, and centrifuging at 8,210 × *g* for 10 min. Samples were analyzed immediately or stored at −20°C until use. LR/pSP-Leptin-3×FLAG samples were prepared by centrifuging 1.5 ml of culture (1 min at 21,130 × *g*), after which we collected the supernatant. Protein from LR/pSP-Leptin-3×FLAG supernatants was precipitated as previously described (84). Samples were loaded onto Bolt 4 to 12% bis-Tris Plus gels (Life Technologies) and transferred onto an iBlot nitrocellulose membrane (Thermo Scientific). The membrane was washed for 1 h in Tris-buffered saline plus Tween 20 (TBST) and 0.5% (wt/vol) milk (blocking buffer) and then hybridized at 4°C for ∼16 h with rabbit anti-Flag antibody (catalog number PA1-984B; Thermo Scientific) diluted 1:500 in blocking buffer. Following three washes with TBST for 5 min each time, horseradish peroxidase-conjugated secondary antibody (anti-rabbit immunoglobulin) was diluted 1:1,000 in blocking buffer and incubated with the membrane for 2 h. Following another three washes with TBST, Clarity Western enhanced chemiluminescence substrate (Bio-Rad) was added and the mixture was incubated with the membrane for 5 min before imaging for chemiluminescence (Bio-Rad Chemi-Doc Touch imaging system).

**(ii) ELISA.** Leptin ELISA (R&D Systems) was performed as suggested by the manufacturer. To measure the amount of intracellular leptin from an overnight cell culture, we processed samples from LR/pLeptin in a manner identical to that described above for preparing samples for Western blotting, while bacterial supernatant samples from the mitomycin C induction experiment were harvested by centrifugation (5 min, 21,130 × *g*), followed by filter sterilization with 0.22-μm-pore-size filters (Millipore). For the mitomycin C induction experiment, total leptin was measured by combining the supernatant with the cell lysate sample. Percent leptin release was calculated by comparing the amount of leptin in the supernatant to the total amount. The final optical density was measured with a microplate reader (450 nm/570 nm; SpectraMax Plus 384; Molecular Devices) within 30 min of the completion of the ELISA. A standard curve was generated using JMP software to calculate leptin concentrations.

### Quantification of bacteria and bacteriophages.

For mitomycin C induction, overnight (∼16-h) cultures were diluted to an OD_600_ of 0.1, and at an OD_600_ of 0.3, mitomycin C was added (0.5 μg/ml; Sigma-Aldrich). Samples were harvested every hour postinduction for 5 h to determine the number of CFU and PFU per milliliter. For analysis of the number of PFU per milliliter, cells were centrifuged (21,130 × *g* for 1 min) and the supernatants were filter sterilized (pore size, 0.22 μm; Millipore). As a lytic host, we used L. reuteri LRΔΦ1ΔΦ2Δ*attB1ΔattB2* (VPL4090) (55), which was prepared as follows: an ∼16-h culture of lytic host was centrifuged at 3,200 × *g* for 5 min and washed once in an equal volume with phage diluent (16 mM MgSO_4_ and 20 mM Tris-Cl, pH 7.5, in distilled H_2_O), followed by resuspension in phage diluent to an OD_600_ of 2.0. Subsequently, we added 10 mM CaCl_2_ to the bacterial suspension. We mixed an equal volume of the lytic host suspension and phage samples (200 μl each) in a 15-ml conical tube and incubated the mixture at 37°C for 1 h. We added 3 ml of 0.2% (wt/vol) agarose harboring 10 mM CaCl_2_, which was gently inverted three times, and poured the mixture onto MRS agar supplemented with 10 mM CaCl_2_, followed by 15 h of incubation at 37°C.

### Plasmid stability assay.

Overnight (∼16-h) cultures of LRΔ*thyA*::*rpoB*(H488R) harboring pLeptin-ThyA, pCtl-ThyA, or pLeptin were diluted to 0.1% in MRS medium without antibiotic. Following 8 h of incubation, serial dilutions from each culture were plated onto plain MRS medium and MRS medium plates harboring 5 μg/ml erythromycin (MRS-Em) for cell viability counts. Cells were passaged twice a day in MRS medium with or without Em for ∼100 generations. Plasmid stability was assessed approximately every 7 generations by calculating the ratio of the number of colonies recovered on MRS-Em plates and the total amount of viable cells recovered on MRS medium plates without antibiotic selection.

### Statistics.

Data representation was performed using DataGraph (version 4.3) software (Visual Data Tools, Inc., Chapel Hill, NC, USA). Statistical comparisons were performed using a paired *t* test, one-way analysis of variance, and Tukey’s honestly significant difference test (HSD) (JMP Pro software, version 11.0.0). Three biological replicates were performed for all *in vitro* studies. All samples were included in the analyses, and experiments were performed without blinding.

### Accession number(s).

The codon-optimized sequence for leptin is available in GenBank under accession number MK297322.
